# Social and Non-Social Cognitive Enhancement in Cocaine Users—A Closer Look on Enhancement Motives for Cocaine Consumption

**DOI:** 10.3389/fpsyt.2020.00618

**Published:** 2020-06-30

**Authors:** Ann-Kathrin Kexel, Matthias Vonmoos, Katrin H. Preller, Lea M. Hulka, Erich Seifritz, Boris B. Quednow

**Affiliations:** ^1^ Experimental and Clinical Pharmacopsychology, Department of Psychiatry, Psychotherapy, and Psychosomatics, Psychiatric Hospital of the University of Zurich, Zurich, Switzerland; ^2^ Department of Psychiatry, Psychotherapy, and Psychosomatics, Psychiatric Hospital of the University of Zurich, Zurich, Switzerland; ^3^ Neuroscience Center Zurich, University of Zurich and Swiss Federal Institute of Technology Zurich, Zurich, Switzerland

**Keywords:** cocaine, stimulants, neuroenhancement, cognitive enhancement, social enhancement, emotion recognition, perspective-taking, cognition

## Abstract

**Background:**

Cognitive disturbances of chronic cocaine users (CU) have been repeatedly investigated. However, it is yet unknown how CU using cocaine for cognitive or social enhancement differ from stimulant-naïve controls and CU that do not have these motives. More precisely, we assumed that CU with an enhancement motive self-medicate deficits in specific cognitive abilities, i.e., they use cocaine to enhance their performance in either social (social motive) or non-social cognitive situations (cognitive motive).

**Methods:**

Forty-two CU were categorized according to their motives for cocaine consumption into social and non-social motive groups as well as cognitive and non-cognitive motive groups, respectively. Subsequently, CU motive groups were compared to 48 stimulant-naïve controls in their social and non-social cognitive functioning applying a comprehensive neuropsychological test battery.

**Results:**

The social motive group showed deficits in cognitive empathy compared to controls (Cohen’s *d* = 0.65) and the non-social motive group (*d* = 0.60). No mentionable effects were found for emotional empathy and Theory-of-Mind. Cognitive and non-cognitive motive groups both showed general cognitive deficits but with different patterns of impairments compared to controls: the cognitive motive group had deficits mainly in working memory (*d* = 0.84) and declarative memory (*d* = 0.60), whereas the non-cognitive motive group also had deficits in working memory (*d* = 0.61) but additionally in executive functions (*d* = 0.67). For the domains declarative memory and executive functions, the respective other CU group displayed intermediate performance.

**Conclusions:**

This study demonstrates that cocaine is partially instrumentalized by CU with specific enhancement motives to counteract related cognitive impairments.

## Introduction

It has been consistently demonstrated, that cocaine users (CU) show broad cognitive impairments spanning from basic functions such as attention and working memory to more complex abilities such as executive functions, social cognition, and social decision-making (1–8). A recent study suggested that 30% of dependent and 12% of recreational users displayed clinically relevant global cognitive impairment (2). While dependent users showed the strongest deficits in working memory and executive functions, recreational users displayed the strongest effect sizes in attention and memory functions (2). Regarding social cognition, CU generally show impaired basic emotion recognition from faces, specifically regarding fear and anger (9–12). Additionally, dependent and even recreational CU revealed moderate deficiencies in emotional empathy (4) as well as in emotion recognition from voices (13). However, only dependent CU showed impaired mental and emotional perspective-taking (Theory-of-Mind, ToM) (4). Finally, CU demonstrated reduced prosocial behavior in social interaction tasks (3) and were less rewarded by social feedback (14). Notably, longitudinal data recently suggested that both cognitive and social cognitive impairments are partially drug-induced and can be improved to a certain extent with decreasing cocaine consumption (15, 16).

Given these relatively broad cognitive deficits and the potential of cocaine to boost cognitive functions acutely (17), one might assume that at least some CU instrumentalize cocaine to self-medicate cognitive impairments that can be either preexisting or induced by chronic cocaine consumption (15, 16, 18). However, the association between cognitive impairment and pharmacological cognitive enhancement (PCE) with cocaine in CU has not been investigated yet. PCE has been defined as the use of psychoactive drugs aiming at “*improving cognition and everyday performance in individuals who suffer from impaired cognition due to brain injury or neuropsychiatric disorders*” [(19), p. 229]. Another definition—mostly employed by bioethicists—is that “*cognitive enhancement refers to the improvement of cognitive ability in normal healthy individuals*” also by pharmacological means [(20), p. 95]. The authors prefer the first definition, as it is broader and therefore includes self-medication of cognitive deficits in illegal substance use populations.

Non-medical use of stimulants for PCE purposes is sometimes practiced by healthy individuals (21, 22). In healthy individuals, the most commonly used stimulant drug for PCE is the medical stimulant methylphenidate (23). However, it has been recently shown in a representative Swiss sample that also illegal stimulants such as street amphetamine and cocaine are used for non-medical PCE, i.e., to increase attention, concentration and memory, even though less frequently compared to recreational purposes (24). Nonetheless, in this study 11.6% of participants who reported cocaine use indicated its use for PCE (24). Importantly, street amphetamine and cocaine as well as medical stimulants such as methylphenidate all share vigilance- and motivation-increasing effects, which are mediated by elevating postsynaptic catecholamine levels (23, 25). To shed further light on the relationship between PCE and illegal substance use, this study focuses on the illicit stimulant cocaine and its use as a cognitive enhancer. As we have shown previously, students reporting non-medical use of methylphenidate for PCE purposes showed superior cognitive performances (off drug) when compared to students not employing such strategies (26). It could therefore also be possible that CU, who apply cocaine for PCE, perform as well as stimulant-naïve controls or that they are less cognitively impaired than CU without PCE motives.

There is currently little knowledge on the reasons for cocaine use, specifically if CU employ PCE strategies in order to compensate for their non-social and social cognitive deficits. If this is the case, this knowledge has important implications for the treatment of cocaine addiction as the PCE motive needs to be addressed and alternatives to cocaine use for PCE have to be identified and implemented. Furthermore, it is unknown if a PCE cocaine use motive is associated with specific cognitive deficits in comparison to stimulant-naïve controls and CU who do not employ PCE strategies. We therefore aimed at characterizing the cognitive profile of CU that reported using cocaine for PCE. Specifically, we asked CU for their motives for cocaine consumption and identified two CU subgroups. CU with a social motive took cocaine to enhance their functioning in social situations and CU with a cognitive motive aimed at better performing in demanding non-social cognitive situations. We hypothesized that CU with a social motive for cocaine consumption have deficits in social cognition and, thus, use cocaine for social PCE. We further expected CU with a cognitive motive for cocaine consumption to have cognitive impairments and thus to use cocaine for non-social PCE.

## Method

### Participants

The sample of 48 stimulant-naïve healthy controls and 42 chronic CU derives from the follow-up assessment of the longitudinal *Zurich Cocaine Cognition Study* (ZuCo^2^St) in which 132 individuals participated (for details please see **Supplementary Methods**). Data of this sample has already been reported in other publications from our group but with different outcome measures or research questions (13, 15, 16, 27). General inclusion criteria at the first assessment were: age between 18 and 60 years, German language proficiency, no current or previous severe medical diseases, neurological disorders or head injuries, no family history of Axis I disorders, no current intake of medication affecting the central nervous system and no regular cannabis consumption. Specific inclusion criteria for CU were cocaine abuse or dependence according to DSM-IV, cocaine as the primary used illegal drug and a current abstinence duration of <6 months. Further exclusion criteria for CU were history of opioid use, polytoxic substance use and previous or current DSM-IV Axis I psychiatric disorders with exception of cocaine, nicotine, cannabis, and alcohol abuse/dependence, attention-deficit/hyperactivity disorder and a previous depressive episode. Specific exclusion criteria for controls were previous or current DSM-IV Axis I psychiatric disorders with exception of nicotine dependence, regular illegal drug use (lifetime use more than 15 occasions for each drug with exception of recreational cannabis use). The study was approved by the Ethics Committee of the Canton Zurich (No. E-14/2009). All participants provided written informed consent in accordance with the Declaration of Helsinki and received monetary compensation for their participation.

### Group Assignment

*Social motive group assignment:* At follow-up CU filled out a questionnaire with ten predefined motives for cocaine consumption and indicated how often they used cocaine to ulfill this motive on a 5-point Likert scale ranging from “never” (1) to “always” (5). In order to identify participants with a social motive, the mean over the three items characterizing a social motive (“I use cocaine to go out”, “to establish contacts more easily”, “to flirt better”) was calculated. CU were then categorized according to a median split on the mean of the three social motives into a social motive (SoM; *Mdn >* 1.83, *n* = 21) and a non-social motive group (NoSoM; *Mdn ≤* 1.83, *n* = 21).

*Cognitive motive group assignment:* In order to examine CU with a cognitive motive, participants were divided according to their rating of the item “I use cocaine to increase my performance (e.g., at work)” into a cognitive motive group (CoM; rating ≥ 2, *n* = 19) and a non-cognitive motive group (NoCoM; rating = 1, *n* = 23).

### Clinical and Substance Use Assessment

Trained psychologists conducted the Structured Clinical Interview for DSM-IV Axis I disorders [SCID-I; (28)]. Additionally, participants carried out the DSM-IV self-rating questionnaire for Axis II personality disorders [SCID-II; (29)]. Because cocaine use was linked to higher ratings on antisocial and narcissistic personality disorder (PD) in this sample at the baseline measurement (4), scores for antisocial and narcissistic PD are reported here. Furthermore, participants filled out the Beck Depression Inventory [BDI; (30)] and the Attention-Deficit/Hyperactivity Disorder (ADHD) Self-Rating Scale [ADHD-SR; (31)] to control for symptoms of depression and ADHD. The *Mehrfachwahl-Wortschatz-Intelligenztest* (32), a standardized German vocabulary test, was used to estimate premorbid verbal intelligence (verbal IQ).

Subjective drug use was quantified with the standardized Interview for Psychotropic Drug Consumption [IPDC; (33)]. Furthermore, in order to objectively assess substance use in the time prior to the test session, hair samples were collected from all participants and analyzed with liquid chromatography-tandem mass spectrometry [LC-MS/MS; for details see (2)]. All participants were asked to abstain from illegal drug use for at least 72 h and from alcohol use 24 h prior to the assessment. Urine toxicology screenings by means of semi-quantitative enzyme multiplied immunoassays [for details see (2)] allowed controlling for compliance with these instructions. Current cocaine craving was measured with the brief version of the Cocaine Craving Questionnaire [CCQ; (34)] and severity of nicotine dependence was assessed with the Fagerström Test of Nicotine Dependence (35).

### Social Cognition

#### Multifaceted Empathy Test (MET)

The MET is a computerized test assessing cognitive (CE) and emotional empathy by evaluating 40 photographs showing people in different positive and negative emotional situations (36). CE was estimated by inferring the mental state of the depicted person by choosing the correct emotion out of four response-alternatives. Emotional empathy was separately evaluated by explicit emotional empathy (EEE), a rating of participants’ empathic concern, and implicit emotional empathy (IEE), a rating of participants’ arousal, on a 9-point Likert scale. We calculated a global emotional empathy domain score (EES) by averaging participants’ scores for EEE and IEE.

#### Movie for the Assessment of Social Cognition (MASC)

The MASC is a 15-min video-based task assessing ToM by asking participants about the video characters’ mental states, hence their feelings, thoughts, and intentions (37). Participants’ are presented with four response-alternatives, one representing the correct answer and three distractors representing each three different types of mistakes: (1) non-mental state inferences, the situation is explained by physical causation (no-ToM), (2) insufficient mental state inferences (undermentalizing, reduced ToM), and (3) excessive mental state inferences (overmentalizing, too much ToM). Based on Wunderli et al. (38), we created a global cognitive empathy domain score (CES) by averaging the MET CE score and the number of correct answers in the MASC after z-transforming them on the means and standard deviations of the control group.

### Cognition

In order to assess cognitive performance participants completed the *Rapid Visual Information Processing*, *Spatial Working Memory*, and *Paired Associates Learning* tasks from the Cambridge Neuropsychological Test Automated Battery (CANTAB^1^) as well as the German version of the Rey Auditory Verbal Learning Test (39) and the Letter Number Sequencing Test (40). Following previous publications from our group investigating cognition in substance users (2, 15, 41, 42), 13 predefined cognitive test parameters were incorporated into the four cognitive domains: attention, working memory, declarative memory, and executive functions [for details see (15, 41)] after z-transforming them on the means and standard deviations of the control group. These four domains were further compiled into a global cognitive index (GCI). In order to avoid alpha-error accumulation, analyses were focused on the four domains and the GCI.

### Statistical Analysis

Statistical analyses were performed with IBM SPSS Statistics 25.0 for Windows. To assess the hypothesized deficits in social cognition for CU of the SoM group and the hypothesized cognitive impairments for CU of the CoM group, we conducted analyses of covariance (ANCOVA) and included age and verbal IQ as covariates (43). Sidak-corrected post-hoc comparisons were carried out where appropriate. The significance level was set at *p <* 0.05 (two-tailed). Cohen’s *d* effect sizes were calculated using the means and pooled standard deviations and can be interpreted with Cohen’s convention of small (0.2), medium (0.5), and large (0.8) effects (44). For more information on additional statistical analyses, please see the **Supplementary Methods**.

## Results

### Social Cognitive Enhancement

#### Demographic Characteristics and Substance Use

Controls, NoSoM and SoM group did not significantly differ in age and sex distribution (**Table 1**). However, the groups were significantly different in years of education and verbal IQ, indicating fewer years of education and a marginally lower verbal IQ for the NoSoM group compared to controls and marginally fewer years of education for the NoSoM compared to the SoM group. Both CU groups scored significantly higher on the BDI and ADHD-SR than controls but did not differ from each other concerning their craving for cocaine.

**Table 1:** Demographic data and cocaine use information for controls and cocaine users in the analysis of social cognitive enhancement.

	Controls (*n* = 48)	NoSoM (*n* = 21)	SoM (*n* = 21)	Value	df/df_err_	*p*
Sex (m/f) (*n*)	32/16	16/5	17/4	*χ^2^* = 1.70^a^	2	0.427
Age	31.35 (8.73)	35.48 (9.41)	30.10 (6.52)	*F* = 2.46^b^	2/87	0.092
Verbal IQ	107.58 (10.04)	101.62 (6.89)†	103.38 (11.17)	*F* = 3.25^b^	2/87	**0.044**
Years of education	10.76 (1.83)	9.57 (1.54)*	10.71 (1.65)‡	*F* = 4.31^c^	2/44.64	**0.019**
ADHD-SR sum score	7.69 (5.19)	12.81 (5.99)**	14.86 (10.02)*	*F* = 8.96^c^	2/35.99	**0.001**
BDI sum score	2.33 (3.27)	7.67 (7.54)*	8.81 (10.52)*	*F* = 8.04^c^	2/29.79	**0.002**
Narcissistic PD	2.07 (1.68)	3.05 (2.11)	5.15 (3.22)**‡	*F* = 8.83^c^	2/33.43	**0.001**
Antisocial PD	2.76 (2.06)	4.15 (3.00)	4.90 (3.96)	*F* = 3.81^c^	2/32.28	**0.033**
Cocaine						
Dependence (y/n) (*n*)	–	9/12	5/16	*χ^2^* = 1.71^a^	1	0.190
Times/week^g,h^	–	0.46 (0.00–2.50)	0.46 (0.11–2.83)	*U* = 263.50^e^		0.278
Grams/week^g,h^	–	0.46 (0.00–11.25)	0.52 (0.13–2.67)	*U* = 238.50^e^		0.650
Years of use	–	11.78 (5.11)	7.56 (5.94)	*t* = 2.47^d^	40	**0.018**
Age of onset	–	24.10 (7.62)	22.60 (4.25)	*t* = 0.79^f^	31.35	0.437
Last consumption (days)^h^	–	8.00 (2.00–182.40)	5.00 (2.00–91.20)	*U* = 171.50^e^		0.216
Cumulative lifetime dose (grams)^h^	–	1,076.09 (90.87–28,103.25)	244.16 (30.42–3,361.96)	*U* = 111.00^e^		**0.006**
Cocaine craving	–	17.24 (10.05)	19.00 (9.61)	*t* = −0.58^d^	40	0.565
Urine toxicology (neg/pos)	48/0	13/8	14/7	*χ^2^* = 20.74^a^	2	**<0.001**
Hair analysis (pg/mg)						
Cocaine_total_^h,i^	–	10,155.00 (1,253–290,250)	5,010.00 (908–64,750)	*U* = 146.00^e^		0.061
Cocaine^h^	–	8,050.00 (1,110–200,000)	4,200.00 (790–59,500)	*U* = 148.00^e^		0.068
Benzoylecgonine^h^	–	1,950.00 (125–84,000)	670.00 (100–9,750)	*U* = 133.00^e^		**0.028**
Norcocaine^h^	–	210.00 (18–6,250)	90.00 (13–725)	*U* = 133.00^e^		**0.028**
Cocaethylene^h^	–	340.00 (0–9,200)	205.00 (0–5,000)	*U* = 196.00^e^		0.538

Significant p-values are shown in bold. Means and standard deviation of means in parenthesis. ADHD-SR, ADHD self-rating scale; BDI, Beck Depression Inventory. ^a^χ^2^ test (across all groups/cocaine users only) for frequency data. ^b^ANOVA (across all groups, with Sidak post-hoc tests vs. controls: ^†^p < 0.10). ^c^Welch’s ANOVA (across all groups, with Games-Howell post-hoc tests vs. controls: *p < 0.05; **p < 0.01; vs. NoSoM: ^‡^p < 0.10). ^d^Independent t-test (cocaine users only). ^e^Mann–Whitney U test (cocaine users only). ^f^Welch’s t-test (cocaine users only). ^g^Average use during the last six months. ^h^Median (range) is reported. ^i^Cocaine_total_ (= cocaine + benzoylecgonine + norcocaine) as a more robust parameter (45).

CU of the SoM group have been using cocaine for a shorter period of time (years of use) and reported less cumulated lifetime dose of cocaine than CU of the NoSoM group (**Table 1**). Further drug reports and hair analyses of both CU groups for other drugs including alcohol revealed a clear preference for cocaine over other substances. For more drug use details see **Table S1** in the **Supplementary Material**.

#### Social Cognition

**Figure 1:**
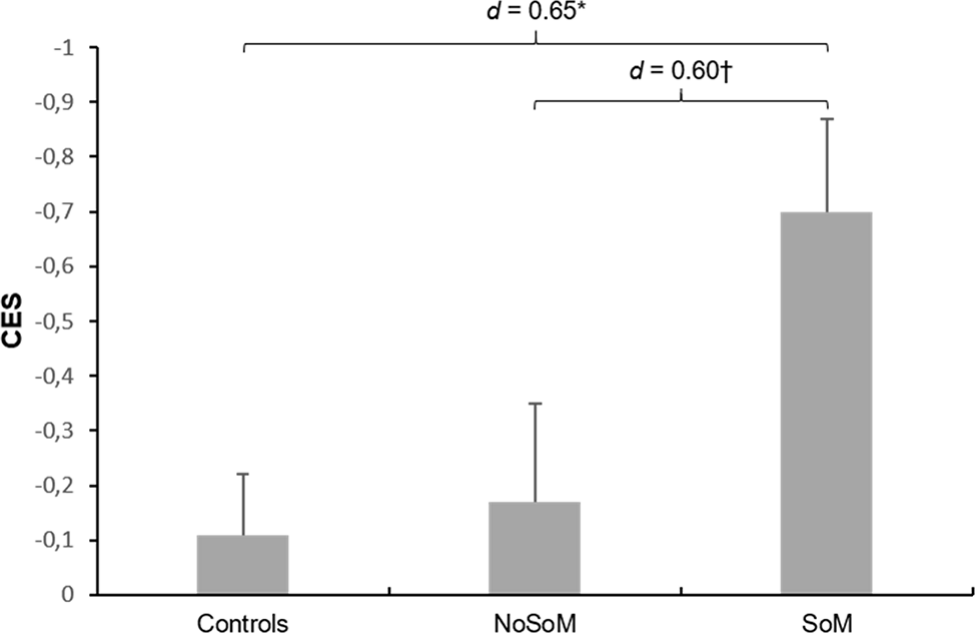
Mean z-scores and standard errors for the Cognitive Empathy Score (CES) in controls and cocaine users with (SoM) and without a social enhancement motive (NoSoM). Values adjusted for verbal IQ and age. Sidak post-hoc tests vs. controls: *p < 0.05; vs. NoSoM: ^†^p < 0.10. Cohen’s d effect sizes for group comparisons are shown on top of the bars.

ANCOVAs controlled for age and verbal IQ showed that controls and CU groups did not significantly differ in EES (*F*(2, 85) = 1.06, *p* = .351). However, there was a significant main effect for group in the global CES (*F*(2, 85) = 4.43, *p* = .015, **Figure 1**). Sidak-corrected post-hoc comparisons revealed that the SoM group performed significantly worse than the control group (*p* < .05, *d* = 0.65) and on a trend-level also worse than the NoSoM group (*p* = .091, *d* = 0.60) with moderate effect sizes, respectively.

**Figure 2:**
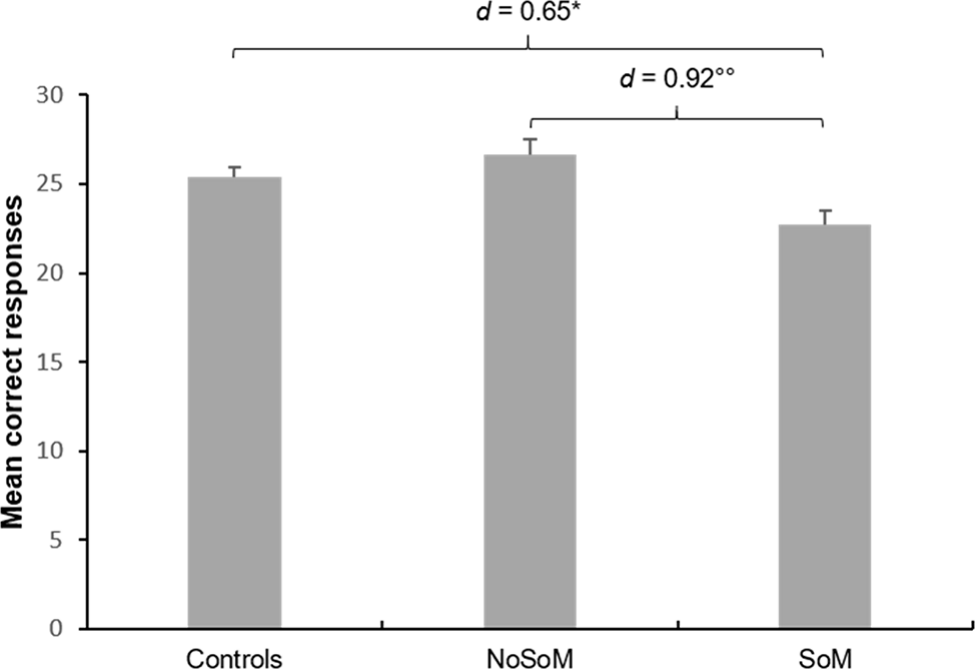
Mean correct responses and standard errors for cognitive empathy in the Multifaceted Empathy Test (MET) of controls and cocaine users with (SoM) and without a social enhancement motive (NoSoM). Values adjusted for verbal IQ and age. Sidak post-hoc tests vs. controls: *p < 0.05; vs. NoSoM: ^°°^p < 0.01. Cohen’s d effect sizes for group comparisons are shown on top of the bars.

Further analyses on the individual scores of the MET and MASC revealed that the effect in the CES was mainly driven by an inferior performance of individuals of the SoM group in CE in the MET (group: *F*(2, 85) = 6.25, *p* = .003, **Figure 2**). Sidak-corrected post-hoc tests indicated significantly fewer correct responses for the SoM group compared to controls (*p* = .020, *d* = 0.65) and to the NoSoM group (*p* = .004, *d* = 0.92) with moderate and strong effect sizes, respectively. There were no group differences for EEE and IEE (for details see **Table S2** in the **Supplementary Material**). An ANCOVA controlling for age and verbal IQ did not reveal significant group differences in the number of wrong answers in the MASC (*F*(2, 84) = 2.23, *p* = .114) but small effect sizes (*d* = 0.40–0.44) indicate that both CU groups made slightly more errors in ToM than controls. Descriptive statistics for social cognitive domain scores, MET, and MASC can be found in **Table S2** in the **Supplementary Material**.

As ADHD is an important covariate when analyzing cognition and social cognition in CU (2, 4, 41), we excluded participants with a suspected diagnosis of ADHD according to the ADHD-SR (*n* = 8 CU) and repeated the analyses. Exclusion of CU with ADHD altered the result for the global CES as this effect no longer remained significant. However, the effect for CE in the MET persisted even after exclusion of subjects with ADHD. Furthermore, to exclude the possibility that our findings were driven by more cumulated lifetime cocaine consumption (2, 15, 16) in the SoM group, we repeated the ANCOVAs adjusted for age and verbal IQ for the domain, the MET, and the MASC scores only comparing CU groups and additionally included ln-transformed cumulated lifetime dose of cocaine. This did not change the results. In an exploratory analysis, we additionally investigated the performance in the neurocognitive domains in the social cognitive enhancement groups. These results can be found in **Table S2** in the **Supplementary Material**.

### Non-Social Cognitive Enhancement

#### Demographic Characteristics and Substance Use

Controls and CU of the NoCoM and CoM group did not significantly differ in age, sex distribution, and years of education (**Table 2**). However, the groups were significantly different in verbal IQ, indicating a marginally lower IQ for the CoM group compared to controls. Both CU groups scored significantly higher on the BDI and ADHD-SR than controls but did not differ from each other concerning their craving for cocaine.

**Table 2:** Demographic data and cocaine use information for controls and cocaine users in the analysis of non-social cognitive enhancement.

	Controls (*n* = 48)	NoCoM (*n* = 23)	CoM (*n* = 19)	Value	df/df_err_	*p*
Sex (m/f) (*n*)	32/16	18/5	15/4	*χ^2^* = 1.59^a^	2	0.453
Age	31.35 (8.73)	34.61 (9.89)	30.58 (5.79)	*F* = 1.48^b^	2/87	0.234
Verbal IQ	107.58 (10.04)	103.04 (7.83)	101.84 (10.84)†	*F* = 3.15^b^	2/87	**0.048**
Years of education	10.76 (1.83)	10.04 (1.72)	10.26 (1.66)	*F* = 1.45^b^	2/87	0.240
ADHD-SR sum score	7.69 (5.19)	13.83 (6.64)**	13.84 (10.00)*	*F* = 9.42^c^	2/34.38	**0.001**
BDI sum score	2.33 (3.27)	8.57 (9.43)*	7.84 (8.82)*	*F* = 7.68^c^	2/29.20	**0.002**
Narcissistic PD	2.07 (1.68)	3.96 (2.51)**	4.29 (3.41)*	*F* = 7.58^c^	2/30.71	**0.002**
Antisocial PD	2.76 (2.06)	4.61 (3.45)†	4.41 (3.64)	*F* = 3.82^c^	2/30.78	**0.033**
Cocaine						
Dependence (y/n) (*n*)	–	6/17	8/11	*χ^2^* = 1.20^a^	1	0.273
Times/week^g,h^	–	0.46 (0.04–1.50)	0.69 (0.00–2.83)	*U* = 302.00^e^		**0.034**
Grams/week^g,h^	–	0.46 (0.06–2.00)	0.81 (0.00–11.25)	*U* = 283.50^e^		0.100
Years of use	–	11.17 (5.96)	7.86 (5.38)	*t* = 1.87^f^	40	0.069
Age of onset	–	23.95 (7.12)	22.61 (4.79)	*t* = 0.70^f^	40	0.489
Last consumption (days)^h^	–	8.00 (2.00–121.60)	5.00 (2.50–182.40)	*U* = 161.00^e^		0.145
Cumulative lifetime dose (grams)^h^	–	633.26 (30.42–28,103.25)	360.20 (36.41–6,603.22)	*U* = 208.00^e^		0.791
Cocaine craving	–	15.26 (5.60)	21.58 (12.46)	*t* = -2.05^f^	23.967	0.052
Urine toxicology (neg/pos)	48/0	16/7	11/8	*χ^2^* = 21.59^a^	2	**<0.001**
Hair sample (pg/mg)						
Cocaine_total_^h,i^	–	5,340.00 (953–290,250)	7,390.00 (908–202,035)	*U* = 235.00^e^		0.677
Cocaine^h^	–	4,500.00 (790–200,000)	6,000.00 (790–170,000)	*U* = 241.00^e^		0.570
Benzoylecgonine^h^	–	1,050.00 (115–84,000)	875.00 (100–31,000)	*U* = 224.00^e^		0.889
Norcocaine^h^	–	135.00 (13–6,250)	150.00 (15–1,035)	*U* = 208.50^e^		0.800
Cocaethylene^h^	–	305.00 (0–9,200)	170.00 (0–8,550)	*U* = 178.00^e^		0.306

Significant p-values are shown in bold. Means and standard deviation of means in parenthesis. ADHD-SR, ADHD self-rating scale; BDI, Beck Depression Inventory. ^a^χ^2^ test (across all groups/cocaine users only) for frequency data. ^b^ANOVA (across all groups, with Sidak post-hoc tests vs. controls: ^†^p < 0.10). ^c^Welch’s ANOVA (across all groups, with Games–Howell post-hoc tests vs. controls: *p < 0.05; **p < 0.01). ^d^Welch’s t-test (cocaine users only). ^e^Mann–Whitney U test (cocaine users only). ^f^Independent t-test (cocaine users only). ^g^Average use during the last six months. ^h^Median (range) is reported. ^i^Cocaine_total_ (= cocaine + benzoylecgonine + norcocaine) as a more robust parameter (45).

The CU groups showed similar patterns of cocaine use (**Table 2**). However, the CoM group indicated higher consumption frequency per week. Drug reports and hair analyses for both CU groups again confirmed a clear preference for cocaine over other substances. For more details see **Table S3**.

#### Cognition

**Figure 3:**
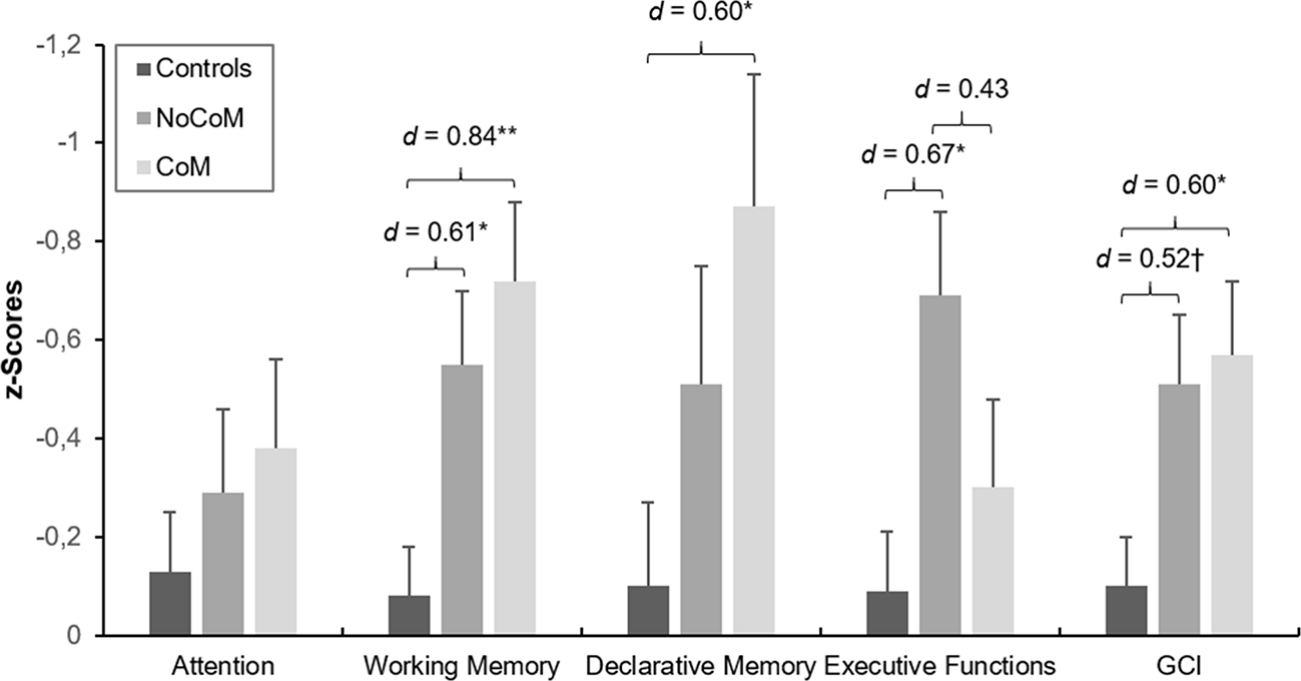
Mean z-scores and standard errors for the four cognitive domains and the Global Cognitive Index (GCI) of controls and cocaine users with (CoM) and without a cognitive enhancement motive (NoCoM). Values adjusted for verbal IQ and age. Sidak post-hoc tests vs. controls: ^†^p < 0.10; *p < 0.05; **p < 0.01. Cohen’s d effect sizes for group comparisons are shown on top of the bars.

ANCOVAs controlled for age and verbal IQ revealed significant group effects in the GCI (*F*(2, 85) = 4.63, *p* = .012, **Figure 3**) and the domains working memory (*F*(2, 85) = 6.76, *p* = .002), declarative memory (*F*(2, 85) = 3.18, *p* = .047), and executive functions (*F*(2, 85) = 4.13, *p* = .002). No significant effect was found for attention (*F*(2, 85) = 0.73, *p* = .485). Sidak-corrected post-hoc comparisons for the GCI demonstrated that only the CoM group performed significantly worse than controls (*p* = .034; *d* = 0.60). The effect for the NoCoM group was only marginally significant (*p* = .059; *d* = 0.52). However, both effects had moderate effect sizes indicating cognitive deficits in CU in general. Regarding the working memory domain, both, the CoM (*p* = .004; *d* = 0.84) and NoCoM group (*p* = .037; *d* = 0.61), performed worse than controls, with the CoM group showing a strong, and the NoCoM group showing a moderate effect size. Concerning declarative memory, only the CoM group showed inferior performance compared to controls (*p* = .048, *d* = 0.60), whereas the NoCoM group showed weaker performance in executive functions (*p* = .015; *d* = 0.67). Both effects had a moderate effect size. Analyses of the individual test parameters underlying the cognitive domains are presented in **Table S4**.

We again excluded subjects with a suspected ADHD and repeated ANCOVAs on the GCI and the cognitive domains. However, this did not change the overall results. We also repeated the analyses only with CU and included ln-transformed cumulated lifetime dose of cocaine. This again did not change the results. In an exploratory analysis, we analyzed social cognition in the non-social cognitive enhancement groups. Results can be found in **Table S5** in the **Supplementary Material**.

## Discussion

We investigated the cognitive profile of CU with a social motive who occasionally take cocaine to enhance their performance in social situations and CU with a cognitive motive who at least sometimes take cocaine to perform better in demanding cognitive situations, e.g., at work.

First, socially motivated CU showed lower cognitive empathy (as reflected by the CES) compared to controls (*d* = 0.65) and, on a trend-level, also compared to CU of the NoSoM group (*d* = 0.60). The lower score in the CES for the SoM group was mainly driven by reduced cognitive empathy in the MET. We therefore propose that CU with a social motive for cocaine consumption use cocaine partially to counteract deficits in the cognitive aspect of empathy. Contrary to the baseline measurement of this sample, we did not find impairments in emotional empathy in the same test (4). Notably, cocaine hair concentrations indicate that the follow-up sample (analyzed here) did not include as many severe users as the baseline sample [compare with (2, 4)] due to a higher drop-out rate within the more severe users. Moreover, although we excluded abstinent (*n* = 7) and non-chronic CU (cocaine hair concentration <500 pg/mg, *n* = 14) at follow-up, the sample still included CU who decreased their cocaine consumption over one year accompanied with improved cognition at follow-up (15, 16). This might explain a lack of findings regarding emotional empathy. Emotional empathy deficits seem to be at least partially drug-induced (16). However, this does not seem to be the case for cognitive empathy and socially motivated CU. Within CU, the inclusion of self-reported cumulated lifetime dose of cocaine did not change the results in social cognition suggesting that these deficits might precede stimulant use. Moreover, the SoM group reported significantly less (*Mdn* = 244.2 g) cumulated lifetime dose of cocaine than the NoSoM group (*Mdn* = 1,076.1 g), additionally supporting that the effect found for cognitive empathy is maybe not induced by previous cocaine use. However, this needs to be verified in further longitudinal studies. Interestingly, Maier et al. (26) examined methylphenidate users that took methylphenidate for PCE and found reduced cognitive empathy on the MET as well, which points to a potential common underlying factor in stimulant users that use the substances for PCE.

Second, we found non-social cognitive impairments in both CU groups as indicated by the GCI, although the comparison between controls and the NoCoM group was only marginally significant (*d* = 0.52). This replicates results from previous studies (1, 5–7) and the baseline measurement (2), although effects were somewhat stronger at the baseline assessment. Both CU groups showed impairments in working memory. However, only CU of the CoM group had additional deficits in declarative memory, whereas only CU of the NoCoM group demonstrated deficits in executive functions. In both cases, the respective other CU group displayed intermediate performance but was not significantly different from either controls or the other CU group. Surprisingly, no differences were found for attention. However, as mentioned before, the follow-up sample was—in the mean—less severely addicted compared to the baseline sample. Given the significant correlation between cognition and cocaine use intensity (2), this likely explains the overall smaller effects in cognition in the present sample of CU. Based on our findings, we propose that CU with a cognitive motive for cocaine consumption use cocaine at least sometimes to counteract deficits, e.g., in working memory functions. Interestingly, they show intermediate performance in executive functions, suggesting that they are likely not (yet) as impaired in this domain as CU without a cognitive motive. This is not surprising as PCE is considered a complex goal-oriented behavior (46). In order to use a substance for PCE, one needs to be able to plan this behavior to get the best outcome. Remarkably, Maier et al. (26) actually found superior functioning of PCE motivated methylphenidate users in executive functions and goal-directed decision-making which points in the same direction. Of note, this study used exactly the same cognitive test battery as applied here.

Additional analyses after exclusion of subjects with a putative diagnosis of ADHD did not change the overall results in cognition whereas some changes occurred for social cognition as the effect in the CES did not remain significant. This is not surprising as we did not observe strong effects in the MASC at baseline (4) where the effect was, additionally, partially driven by individuals with a suspected ADHD diagnosis (41). However, the effect on the cognitive empathy score of the MET persisted. After exclusion of subjects with ADHD, the respective effects in the MET and MASC canceled each other out leading to a non-significant result in the composite score. Nevertheless, we believe that our main results are still valid and meaningful even without considering ADHD, as the motive to enhance social and non-social cognitive performance through cocaine consumption needs to be considered within the entire profile of the CU and often, cocaine use is associated with an ADHD diagnosis (2, 15, 41, 47–49). Notably, CU with ADHD (*n* = 8) appeared more frequently in the enhancement groups with seven CU with ADHD in the SoM (*p* < .001, Fisher’s exact test) and six in the CoM group (*p* < .001, Fisher’s exact test) indicating the use of cocaine as a form of self-medication especially for those CU with a putative comorbid ADHD.

The findings need to be interpreted with the following limitations in mind. First, controls, NoSoM and SoM groups differed with regard to verbal IQ and years of education and controls and CoM group differed with regard to verbal IQ. However, groups were matched for age and sex and we included age and verbal IQ as covariates in the statistical models. We did not include years of education as a covariate as only verbal IQ differed in both investigations and verbal IQ and years of education were moderately correlated with each other (*r* = 0.31, *p* < .01). Second, sample sizes of the CU groups were small and only a few CU utilized cocaine intensively as a social or cognitive enhancer. Therefore, the results need to be replicated in larger samples of CU who instrumentalize cocaine for PCE more regularly.

This study demonstrates that a subgroup of CU who sometimes employ PCE strategies also show social and non-social cognitive deficits. It is therefore conceivable, that these subgroups try to compensate cognitive deficits with cocaine use. This instrumentalization of cocaine use can be considered as a self-medication of pre-existing or cocaine-induced cognitive impairments (or both), which seems to be especially true for CU with a co-morbid ADHD diagnosis. Consequently, existing PCE strategies have important implications for treatment outcomes as these strategies need to be addressed and alternatives to satisfy the motive need to be found. In general, social and non-social cognitive impairments have been proposed to diminish the efficacy of therapeutic interventions (50, 51). Thus, psychotherapeutic but also psychopharmacological approaches were recently suggested to alleviate cognitive deficits in CU in order to improve addiction therapy success (51–53). For instance, cognitive enhancement with ADHD (or other) medications has been proposed as a treatment target for cognitive disturbances in cocaine use disorder in the past (53–55). However, contrary to opioid or nicotine dependence, substitution in cocaine use disorder is not yet approved due to unclear evidence from clinical studies (56–58). Nevertheless, substitution with methylphenidate has been proposed to be a safe and effective treatment in CU with comorbid ADHD (57). As our findings indicate that especially CU with comorbid ADHD employ PCE strategies and that cognitive impairments in CU with ADHD are amplified (41), we assume that substitution could be beneficial in these patients. We therefore suggest that attending physicians discuss putative PCE strategies and cognitive impairments with their patients in general, explain the negative long-term consequences of cocaine use (15, 16, 18) and additionally offer PCE with medical drugs as an alternative to CU with comorbid ADHD. This could foster treatment compliance as patients are signaled that their personal goals in using cocaine are respected and met. However, the use of medical stimulants might delay recovery that can be observed after longer periods of abstinence as the reversibility of cocaine-induced cognitive dysfunctions has been proposed to reflect re-adaptation of brain functions and neurotransmitter systems (15).

## Data Availability Statement

The raw data supporting the conclusions of this article will be made available by the authors, without undue reservation.

## Ethics Statement

The studies involving human participants were reviewed and approved by the Ethics Committee of the Canton Zurich. The patients/participants provided their written informed consent to participate in this study.

## Author Contributions

BQ and A-KK had full access to all the data in the study and take responsibility for the integrity of the data and the accuracy of the data analysis. BQ developed the study concept and design. MV, KP, and LH contributed to the acquisition of the data, A-KK and BQ analyzed and interpreted the data. A-KK and BQ drafted the manuscript. All authors contributed to the article and approved the submitted version. ES and BQ obtained funding. MV, KP, LH, and ES contributed to the administrative, technical, or material support. ES and BQ were in charge of supervision.

## Funding

The study was supported by grants from the Swiss National Science Foundation (Grant Nos. PP00P1-123516/1 and PP00P1-146326/1) and the Olga Mayenfisch Foundation. A-KK was financed by a grant of the Swiss National Science Foundation (105319_162639) to BQ. The funders had no role in study design; collection, management, analysis, and interpretation of the data; preparation, review, and approval of the manuscript, and the decision to submit the manuscript for publication.

## Conflict of Interest

The authors declare that the research was conducted in the absence of any commercial or financial relationships that could be construed as a potential conflict of interest.

## Footnotes

1
http://www.cantab.com.
