# Supplementary material for: Social and Non-Social Cognitive Enhancement in Cocaine Users—A Closer Look on Enhancement Motives for Cocaine Consumption
Source: Front Psychiatry. 2020 Jun 30;11:618. doi: 10.3389/fpsyt.2020.00618 (PMC7338788; doi:10.3389/fpsyt.2020.00618)
Supplement: Supplementary file 1 [file DataSheet_1.pdf]

Supplementary Material

1. Supplementary Methods

Methods S1. Recruitment and participant selection.

Participants were recruited at the baseline assessment of the longitudinal Zurich Cocaine Cognition Study ($ZuCo^2St$) through drug prevention and treatment centers, psychiatric hospitals, advertisements in local newspapers, online media, and word of mouth.

132 participants (79 cocaine users, 53 controls) participated in the follow-up assessment of the longitudinal $ZuCo^2St$. Of these, 21 subjects (16 cocaine users, 5 controls) were excluded due to the use of illegal substances, objectively verified by hair analyses, that were not allowed by our exclusion criteria (e.g., opioids or excessive MDMA consumption) or beginning of intake of psychotropic medication (e.g., antipsychotics or antidepressants). Furthermore, additional 21 cocaine users whose hair concentration of cocaine was below cut-off (< 500 pg/mg) were excluded to ensure that only regular cocaine users were included in the final study sample.

Methods S2. Statistical analysis.

Demographic and substance use data were analyzed by means of Pearson's χ^2-test and analyses of variance (ANOVA) followed by Sidak-corrected post-hoc comparisons where appropriate. If data were not normally distributed or the homogeneity of variance assumption was violated, the Kruskal-Wallis-test with Bonferroni-corrected post-hoc comparisons and Welch's ANOVA with Games-Howell-corrected post-hoc comparisons were carried out, respectively. Analyses for CU only were either implemented with independent *t*-tests or, if data were non-normally distributed or showed heterogeneity of variance, with Mann-Whitney *U*-test or Welch's *t*-test, respectively.

Due to the highly skewed distribution of cumulated lifetime cocaine dose (Shapiro-Wilk $W(42)=0.52$, $p<.001$), this variable was ln-transformed for further statistical analyses.

2. Supplementary Tables

Table S1. Pattern and amount of substance use for controls and cocaine users in the analysis of social cognitive enhancement.

	Controls (n = 48)	NoSoM (n = 21)	SoM (n = 21)	Value	df/df_{err}	p
**Nicotine**						
Smoking (n)	40	18	17	χ^2 = 0.17[a]	2	0.918
Cigarettes per day[g, h]	9.85 (8.69)	13.39 (7.09)	16.00 (10.99)	F = 3.09[b]	2/72	0.052
Years of use[h]	12.02 (8.18)	17.69 (7.86)*	12.45 (5.28)	F = 3.70[b]	2/72	**0.030**
FTND sum score[h]	1.93 (2.39)	4.29 (2.37)**	4.25 (2.54)**	F = 8.52[b]	2/70	**<0.001**
**Alcohol**						
Grams/week[g]	104.35 (88.58)	210.62 (240.02)	196.92 (165.09)†	F = 4.43[c]	2/31.17	**0.020**
Years of use	14.00 (8.68)	16.16 (7.09)	12.79 (6.11)	F = 1.02[b]	2/87	0.364
**Cannabis**						
Grams/week[g, i]	0.00 (0.00 – 10.00)	0.00 (0.00 – 20.00)	0.17 (0.00 – 10.00)	H = 5.40[d]	2	0.067
Years of use	4.57 (5.92)	9.36 (11.11)	10.78 (7.27)**	F = 6.69[c]	2/35.55	**0.003**
Last consumption (days)[i]	7.00 (0.50 – 2737.35)	5.00 (0.13 – 212.80)	3.75 (0.50 – 182.40)	H = 1.65[d]	2	0.439
Cumulative lifetime dose (grams)[i]	74.29 (0.00 – 27555.00)	836.63 (0.00 – 27789.36)	728.45 (0.00 – 9705.06)†	H = 6.63[d]	2	**0.036**
Urine toxicology (neg/pos)	42/6	14/7	10/11	χ^2 = 12.50[a]	2	**0.002**
**Amphetamines**						
Grams/week[g, i]	–	0.00 (0.00 – 0.81)	0.00 (0.00 – 0.35)	U = 225.00[e]		0.875
Years of use[i]	0.00 (0.00 – 3.00)	0.00 (0.00 – 13.00)	2.50 (0.00 – 17.00)***	H = 26.31[d]	2	**<0.001**
Last consumption (days)	–	50.40 (36.28)	114.24 (123.90)	t = -1.59[f]	13.99	0.134
Cumulative lifetime dose (grams)[i]	0.00 (0.00 – 0.65)	0.00 (0.00 – 782.15)**	2.40 (0.00 – 260.70)***°°	H = 33.83[d]	2	**<0.001**
Hair sample (pg/mg)[i]	–	0.00 (0.00 – 1090)	0.00 (0 – 2000)	U = 247.00[e]		0.389
**MDMA**						
Pills/week[g, i]	0.00 (0.00 – 0.06)	0.00 (0.00 – 2.07)	0.00 (0.00 – 3.50)***°	H = 22.18[d]	2	**<0.001**

Years of use[i]	0.00 (0.00 – 10.00)	0.00 (0.00 – 15.00)*	3.00 (0.00 – 19.00)***	H = 21.40[d]	2	**<0.001**
Last consumption (days)[i]	121.60 (60.80 – 364.98)	106.40 (14.00 – 364.81)	25.71 (4.00 – 290.60)	H = 2.83[d]	2	0.242
Cumulative lifetime dose (grams)[i]	0.00 (0.00 – 22.00)	2.50 (0.00 – 876.87)**	17.05 (0.00 – 580.41)***	H = 30.50[d]	2	**<0.001**
Hair sample (pg/mg)[i]	0.00 (0 – 143)	0.00 (0 – 20500)	80.00 (0 – 6500)***°	H = 31.29[d]	2	**<0.001**
**GHB**						
Cumulative lifetime dose (pipettes)[i]	–	0.00 (0.00 – 2.00)	0.00 (0.00 – 79.00)	U = 221.00[e]		0.988
**Hallucinogens**						
Cumulative lifetime dose (times)[i]	0.00 (0.00 – 10.00)	1.00 (0.00 – 324.50)*	4.50 (0.00 – 55.00)***	H = 18.87[d]	2	**<0.001**

Significant p-values are shown in bold. Means and standard deviation of means in parenthesis. Abbreviations: FTND: Fagerström Test of Nicotine Dependence. [a] χ^2 test (across all groups/cocaine users only) for frequency data. [b] ANOVA (across all groups, with Sidak post-hoc tests vs. controls: *p<0.05; **p<0.01). [c] Welch's ANOVA (across all groups, with Games-Howell post-hoc tests vs. controls: †p<0.10; **p<0.01). [d] Kruskal-Wallis Test (with Bonferroni post-hoc tests vs. controls: †p<0.10; *p<0.05; **p<0.01; ***p<0.001; vs. NoSoM: °p<0.05; °°p<0.01). [e] Mann-Whitney-U-Test (cocaine users only). [f] Welch test (cocaine users only). [g] Average use during the last six months. [h] Only smokers. [i] Median (range) is reported.

Table S2. Social and general cognition data in the analysis of social cognitive enhancement.

	Controls (*n* = 48)	NoSoM (*n* = 21)	SoM (*n* = 21)	*F*	df/df_{err}	*p*
**Social cognitive domains**						
Emotional empathy score	5.22 (0.22)	4.86 (0.35)	4.65 (0.33)	1.06	2/85	0.351
Cognitive empathy score	-0.11 (0.11)	-0.17 (0.18)	-0.70 (0.17)*‡	4.43	2/85	**0.015**
**MET**						
Explicit emotional empathy	5.36 (0.23)	5.11 (0.35)	4.80 (0.34)	0.96	2/85	0.387
Implicit emotional empathy	5.07 (0.23)	4.62 (0.35)	4.51 (0.34)	1.15	2/85	0.322
Cognitive empathy	25.43 (0.54)	26.64 (0.84)	22.71 (0.81)*°°	6.25	2/85	**0.003**
**MASC**						
ToM total errors[a]	8.83 (0.55)	10.74 (0.86)	10.40 (0.82)	2.23	2/84	0.114
**Cognitive domains**						
Attention	-0.13 (0.12)	-0.51 (0.18)	-0.17 (0.17)	1.59	2/85	0.211
Working memory	-0.07 (0.10)	-0.71 (0.16)**	-0.54 (0.15)*	6.72	2/85	**0.002**
Declarative memory	-0.09 (0.17)	-0.88 (0.26)*	-0.48 (0.25)	3.28	2/85	**0.043**
Executive functions	-0.09 (0.12)	-0.70 (0.18)*	-0.35 (0.17)	3.84	2/85	**0.025**
Global cognitive index	-0.09 (0.10)	-0.70 (0.15)**	-0.39 (0.14)	5.87	2/85	**0.004**

Significant p-values are shown in bold. Means and standard error of the mean in parenthesis. Adjusted for verbal IQ and age. Sidak post-hoc tests vs. controls: *p<0.05; **p<0.01; vs. NoSoM: ‡p<0.10; °°p<0.01. [a] In the MASC, data is missing for one participant of the NoSoM group.

Table S3. Pattern and amount of substance use for controls and cocaine users in the analysis of non-social cognitive enhancement.

	Controls (*n* = 48)	NoCoM (*n* = 23)	CoM (*n* = 19)	Value	df/df_{err}	*p*
**Nicotine**						
Smoking (*n*)	40	19	16	χ^2 = 0.02[a]	2	0.990
Cigarettes per day[f, g]	9.85 (8.69)	14.73 (6.23)	14.57 (11.97)	*F* = 2.69[b]	2/72	0.075
Years of use[g]	12.02 (8.18)	15.88 (8.21)*	14.28 (5.78)	*F* = 1.70[b]	2/72	0.190
FTND sum score[g]	1.93 (2.39)	4.32 (2.34)**	4.21 (2.61)**	*F* = 8.53[b]	2/70	**<0.001**
**Alcohol**						
Grams/week[f]	104.35 (88.58)	215.78 (228.99)†	189.24 (172.94)	*F* = 4.19[c]	2/30.75	**0.025**
Years of use	14.00 (8.68)	15.38 (8.07)	13.39 (4.69)	*F* = 0.38[b]	2/87	0.687
**Cannabis**						
Grams/week[f, h]	0.00 (0.00 – 10.00)	0.00 (0.00 – 5.00)	0.38 (0.00 – 20.00)**°	*H* = 10.48[d]	2	**0.005**
Years of use	4.57 (5.92)	8.77 (10.37)	11.64 (7.80)**	*F* = 7.00[c]	2/35.02	**0.003**
Last consumption (days)[h]	7.00 (0.50 – 2737.35)	5.00 (1.00 – 91.20)	3.75 (0.13 – 212.80)	*H* = 1.71[d]	2	0.426
Cumulative lifetime dose (grams)[h]	74.29 (0.00 – 27555.00)	219.60 (0.00 – 27789.36)	1322.20 (0.00 – 24809.85)**	*H* = 9.98[d]	2	**0.007**
Urine toxicology (neg/pos)	42/6	16/7	8/11	χ^2 = 14.57[a]	2	**0.001**
**Amphetamines**						
Grams/week[f, h]	–	0.00 (0.00 – 0.81)	0.00 (0.00 – 0.35)	*U* = 232.00[e]		0.634
Years of use[h]	0.00 (0.00 – 3.00)	0.00 (0.00 – 13.00)	1.17 (0.00 – 17.00)***	*H* = 21.85[d]	2	**<0.001**
Last consumption (days)[h]	–	121.60 (4.00 – 273.60)	25.70 (4.00 – 364.98)	*U* = 20.50[e]		0.313
Cumulative lifetime dose (grams)[h]	0.00 (0.00 – 0.65)	0.10 (0.00 – 104.28)*	3.27 (0.00 – 782.15)**	*H* = 34.16[d]	2	**<0.001**
Hair sample (pg/mg)[h]	–	0.00 (0 – 1090)	0.00 (0 – 2000)	*U* = 260.00[e]		0.175
**MDMA**						
Pills/week[f, h]	0.00 (0.00 – 0.06)	0.00 (0.00 – 2.07)†	0.00 (0.00 – 3.50)***	*H* = 17.34[d]	2	**<0.001**
Years of use[h]	0.00 (0.00 – 10.00)	0.00 (0.00 – 15.00)*	3.50 (0.00 – 19.00)***	*H* = 23.89[d]	2	**<0.001**

Last consumption (days)[h]	121.60 (60.80 – 364.98)	24.50 (11.00 – 364.81)	121.60 (4.00 – 212.80)	*H* = 2.08[d]	2	0.353
Cumulative lifetime dose (grams)[h]	0.00 (0.00 – 22.00)	1.12 (0.00 – 580.41)*	18.00 (0.00 – 876.87)**	*H* = 31.06[d]	2	**<0.001**
Hair sample (pg/mg)[h]	0.00 (0 – 143)	0.00 (0 – 20500)**	35.00 (0 – 6500)***	*H* = 25.08[d]	2	**<0.001**
**GHB**						
Cumulative lifetime dose (pipettes)[h]	–	0.00 (0.00 – 2.00)	1.00 (0.00 – 79.00)	*U* = 309.00[e]		**0.005**
**Hallucinogens**						
Cumulative lifetime dose (times)[h]	0.00 (0.00 – 10.00)	1.00 (0.00 – 55.14)†	4.50 (0.00 – 324.50)***‡	*H* = 21.47[d]	2	**<0.001**

Significant p-values are shown in bold. Means and standard deviation of means in parenthesis. Abbrevations: FTND: Fagerström Test of Nicotine Dependence. [a] χ^2 test (across all groups/cocaine users only) for frequency data. [b] ANOVA (across all groups, with Sidak post-hoc tests vs. controls: *p<0.05; **p<0.01). [c] Welch's ANOVA (across all groups, with Games-Howell post-hoc tests vs. controls: †p<0.10). [d] Kruskal-Wallis Test (with Bonferroni post-hoc tests vs. controls: †p<0.10; *p<0.05; **p<0.01; ***p<0.001; vs. NoCoM: ‡p<0.10; °p<0.05). [e] Mann-Whitney-U-Test (cocaine users only). [f] Average use during the last six months. [g] Only smokers. [h] Median (range) is reported.

Table S4. Scores of neuropsychological tests.

	Controls (*n* = 48)	NoCoM (*n* = 23)	CoM (*n* = 19)	F^a	df/df_{err}	*p*
**Attention**						
RVP Discrimination performance A‘	0.93 (0.01)	0.92 (0.01)	0.92 (0.01)	0.63	2/85	0.537
RVP Total hits	19.33 (0.59)	18.77 (0.86)	18.23 (0.94)	0.51	2/85	0.605
RAVLT Supraspan trial 1[b]	9.53 (0.33)	8.95 (0.47)	8.95 (0.51)	0.71	2/84	0.496
**Working memory**						
LNST Score	15.43 (0.41)	14.32 (0.59)	13.23 (0.64)*	4.33	2/85	**0.016**
SWM Total errors	14.47 (2.24)	25.60 (3.24)*	24.72 (3.53)†	5.13	2/85	**0.008**
PAL First trial memory score	16.17 (0.45)	15.38 (0.65)	14.63 (0.71)	1.76	2/85	0.179
**Declarative memory**						
RAVLT Learning performance (∑trial 1-5)[1]	63.82 (1.19)	59.50 (1.71)	59.35 (1.86)	3.05	2/84	0.052
RAVLT Adjusted recognition p(A)[b]	0.86 (0.02)	0.85 (0.03)	0.83 (0.03)	0.36	2/84	0.701
RAVLT Delayed recall trial 7[b]	13.51 (0.36)	12.88 (0.52)	12.03 (0.56)	2.42	2/84	0.095
PAL Total errors adjusted	7.97 (1.91)	12.84 (2.77)	16.90 (3.01)*	3.27	2/85	**0.043**
PAL Total trials adjusted	8.14 (0.43)	8.57 (0.62)	9.74 (0.67)	1.97	2/85	0.146
**Executive functions**						
SWM Strategy score	30.12 (0.81)	32.75 (1.17)	30.70 (1.28)	1.67	2/85	0.194
RAVLT Recall consistency (%)[b]	93.01 (1.17)	87.99 (1.67)	90.75 (1.83)	2.95	2/84	0.058

Significant p-values are shown in bold. Means and standard error of the mean in parenthesis. Adjusted for verbal IQ and age. Abbreviations: RVP: Rapid Visual Information Processing; RAVLT: Rey Auditory Verbal Learning Test; LNST: Letter Number Sequencing Test; SWM: Spatial Working Memory; PAL: Paired Associates Learning. [a] ANCOVA (across all groups, with Sidak post-hoc tests vs. controls: †p<0.10; *p<0.05). [b] In the RAVLT, data is missing for one control subject.

Table S5. Social cognition data in the analysis of non-social cognitive enhancement.

	Controls (*n* = 48)	NoCoM (*n* = 23)	CoM (*n* = 19)	*F*	df/df_{err}	*p*
**Social cognitive domains**						
Emotional empathy score	5.21 (0.21)	5.33 (0.31)	4.08 (0.34)*°	4.79	2/85	**0.011**
Cognitive empathy score	-0.10 (0.12)	-0.25 (0.17)	-0.67 (0.18)*	3.44	2/85	**0.037**
**MET**						
Explicit emotional empathy	5.36 (0.22)	5.50 (0.32)	4.29 (0.34)*°	4.21	2/85	**0.018**
Implicit emotional empathy	5.07 (0.22)	5.15 (0.32)	3.86 (0.34)*°	5.05	2/85	**0.008**
Cognitive empathy	25.49 (0.56)	25.89 (0.81)	23.06 (0.88)†‡	3.42	2/85	**0.037**
**MASC**						
ToM total errors	8.84 (0.55)	10.58 (0.81)	10.53 (0.86)	2.19	2/84	0.118

Significant p-values are shown in bold. Means and standard error of the mean in parenthesis. Adjusted for verbal IQ and age. Sidak post-hoc tests vs. controls: †p<0.10; *p<0.05; vs. NoCoM: ‡p<0.10; °p<0.05.
